# Sodium chloride pica causing recurrent nephrolithiasis in a patient with iron deficiency anemia: a case report

**DOI:** 10.1186/s13256-017-1499-5

**Published:** 2017-11-18

**Authors:** Brittany Rogers, Joshua Kramer, Stephanie Smith, Vincent Bird, Eric I. Rosenberg

**Affiliations:** 1 Department of Medicine, Division of General Internal Medicine, College of Medicine, University of Florida, 1329 SW 16th Street, Suite 5140, Gainesville, FL 32610 USA; 2 Department of Clinical and Health Psychology, College of Public Health and Health Professions, University of Florida, Florida, USA; 3 Division of Minimally Invasive Surgery, Department of Urology, College of Medicine, University of Florida, Florida, USA

**Keywords:** Pica, Anemia, Iron deficiency, Nephrolithiasis, Case report

## Abstract

**Background:**

Iron deficiency anemia is a common finding in women of child-bearing age. Pica, or the ingestion of non-food or non-nutritive items, is a well-known manifestation of iron deficiency. A high sodium diet increases risk for nephrolithiasis. We describe the case of a 31-year-old woman with recurrent calcium nephrolithiasis and anemia who ate ice chips as well as spoons of salt daily. Treatment of pica may prove effective in preventing recurrent nephrolithiasis.

**Case presentation:**

A 31-year-old white woman with a past medical history of menorrhagia, anemia, and recurrent calcium nephrolithiasis presented for preoperative evaluation prior to ureterolithotomy. She described a daily pattern of eating continually from a cup of ice chips accompanied by multiple spoons of salt directly out of a salt shaker. These cravings had been present for many years, were bothersome to her, and interfered with her daily life. Laboratory findings revealed hemoglobin of 10.9 g/dL with ferritin of 3 ng/mL. History, physical, and laboratory data were consistent with pica secondary to iron deficiency anemia. She was prescribed orally administered ferrous sulfate 325 mg three times a day with meals. She continues to struggle with the symptoms of pica and orally administered supplementation.

**Conclusions:**

It is important that clinicians consider the possible diagnosis of sodium chloride pica in patients with iron deficiency anemia and recurrent nephrolithiasis. Treatment of anemia and resolution of pica may prove effective in preventing future nephrolithiasis. Specific questioning about pica symptoms in patients with iron deficiency anemia and recurrent nephrolithiasis may be helpful diagnostically and therapeutically.

## Background

Pica is a well-known physical manifestation of nutrient deficiency, most commonly iron. Cravings for non-nutritive food items including sand or clay (geophagia), gravel or stone (lithophagia), ice (pagophagia), and starch (amylophagia) become compulsions that interfere with the patient’s day-to-day life. It has been estimated that pica occurs in as many as 50% of patients with iron deficiency anemia [1]. Pica is most frequently seen in pregnant women, children, and people of low socioeconomic class. Pica has been documented in written history since the time of Hippocrates [2]. The long-term consequences of pica are not well understood; toxicity from metabolic abnormalities arising from elemental and vitamin deficiencies, as well as bowel obstruction, excessive calorie intake, and dental damage have all been observed in the short term [3]. In some patients, pica symptoms may be short lived, but in other patients the symptoms may persist for longer periods undiagnosed and have more adverse health effects.

Sodium chloride pica is a very rare form of pica [4]. In 1985, Shapiro and Linus described a 33-year-old woman with hypertension and history of dysfunctional uterine bleeding who experienced very similar salt cravings and was diagnosed with salt pica secondary to iron deficiency anemia [4]. Her pica symptoms and anemia resolved following treatment with a 4-week course of parenteral iron following failure of 1 month of orally administered iron therapy. A high sodium diet increases risk for nephrolithiasis. In a patient with normal renal function, increased sodium intake is excreted by the kidneys to maintain homeostasis. Increased sodium excretion promotes calcium excretion which facilitates the formation of calcium stones [5].

## Case presentation

A 31-year-old white woman with a past medical history of menorrhagia, anemia, and recurrent calcium nephrolithiasis presented for preoperative evaluation prior to ureterolithotomy. She described a daily pattern of eating continually from a cup of ice chips accompanied by multiple spoons of salt directly out of a salt shaker. These cravings had been present for many years, were bothersome to her, and interfered with her daily life. She noticed an acute worsening of these symptoms following delivery of her last child. She described 5 days of heavy bleeding during her menstrual cycle. She denied chalk, clay, or dirt cravings. She denied lightheadedness, syncope or near syncope, or melena.

On physical examination, her temperature was 36.7 °C, blood pressure was 119/70 mmHg, heart rate was 88 beats per minute, respirations were 20 per minute, and oxygen saturation was 100% on room air. Her body mass index (BMI) was 26.3 kg/m^2^. She was a well-nourished, well-developed woman. A physical examination was negative for stomatitis, glossitis, or pallor. An abdominal examination was negative for splenomegaly. Mentation was appropriate. Mood and affect were normal. Initial laboratory findings including hematology and iron studies are shown in Table 1. Previous stone analysis is shown in Table 2.

**Table 1:** Hematology and iron profile

	Initial consult	3 Months later	Normal range
WBC	7.6	4.8	4.0–10.0 thousand/mm^3^
RBC	4.57	4.26	4.0–5.2 million/mm^3^
Hemoglobin	10.9	9.1	12.0–16.0 g/dL
Hematocrit	33.9	28.0	35.0–45.0%
MCV	74.2	67.4	78.0–100.0 μm^3^
MCH	24.0	21.9	26–34 pg
RDW	14.1%	15.6%	11.0–14.0%
Reticulocyte count	1.2%	--	--
Platelets	286	194	150–450 thousand/mm^3^
Total iron	22 mcg/dL	--	40–190 mcg/dL
TIBC	431 mcg/dL	--	250–450 mcg/dL
Iron saturation	5%	--	11–50%
Ferritin	3 ng/mL	--	10–154 ng/mL
Transferrin	323 mg/dL	--	188–341 mg/dL

*MCH* mean corpuscular hemoglobin, *MCV* mean corpuscular volume *RBC* red blood cells, *RDW* random distribution of red cell width, *TIBC* total iron-binding capacity, *WBC* white blood cells

**Table 2:** Stone analysis

Composition	Percentage
Calcium oxalate monohydrate	55%
Calcium phosphate hydroxyl form	30%
Calcium oxalate dehydrate	7%
Calcium phosphate carbonate	5%
Protein	3%

History, physical, and laboratory data were consistent with pica secondary to iron deficiency anemia. The differential diagnosis for her iron deficiency anemia included principally menorrhagia but consideration was also paid to possible occult gastrointestinal blood loss due to occult peptic ulcer disease, inflammatory bowel disease, parasitosis, malabsorption, or celiac disease. She had no history of diarrhea or abnormal constipation patterns. She had no history of weight loss, abdominal cramping, or pain. She described no international travel and had no history of exposure to untreated well-water. She had no history of nonsteroidal anti-inflammatory drug (NSAID) use.

She was prescribed ferrous sulfate 325 mg administered orally three times a day with meals. She was hospitalized 3 months later for pain related to recurrent kidney stones. At that time, she reported that she had not been the taking the orally administered iron supplementation which had been prescribed. Her anemia worsened, as seen in Table 1.

She was telephoned to follow-up on her current symptoms. She was unable to tolerate the orally administered iron due to symptoms of nausea and dyspepsia. She stated that her pica symptoms continued to be constant throughout the day. She was advised to return to our clinic for further evaluation and to discuss alternative iron supplementation. She has continued to have intermittent adherence to follow-up for her anemia with her primary care physician, limiting further workup and treatment.

## Discussion

It is important that clinicians consider sodium chloride pica in patients with iron deficiency anemia and recurrent nephrolithiasis, because treatment of anemia and resolution of pica may prove effective in preventing future nephrolithiasis. In patients with normal renal function, urine calcium excretion increases 0.6 mmol/day per each 100 mmol/day of excreted sodium [5]. A low sodium diet (less than 100 mEq/day) facilitates proximal tubule sodium and calcium reabsorption and limits calcium excretion into the urine [6]. It has been shown that decreasing sodium intake from 200 to 80 mEq/day can decrease calcium excretion by 100 mg/day (2.5 mmol/day) [7].

Our patient was unable to complete a 24-hour urine collection to confirm our hypothesis and exclude other possibilities. We anticipate this study would have revealed elevated urinary sodium and calcium concentrations. Metabolic evaluation and potassium citrate therapy should be considered for young adult patients with a stone to prevent recurrence. Oxalate seems to be the most significant urinary stone risk factor in this population followed by calcium and uric acid [8]. In addition to each individual risk factor, it appears that supersaturation with the sum of risk factors probably has the highest predictive value. Hypocitraturia should always be considered, confirmed by 24-hour urine; it is present in 30% of patients who are stone formers, more so in men [9]. Young age (18 to 29 years) at first stone presentation was a significant risk factor for stone recurrence, and urinary citrate excretion was an independent risk factor affecting recurrence in this group [10].

Pica is included in the “Feeding and Eating Disorders” section of the *Diagnostic and Statistical Manual of Mental Disorders*, Fifth Edition (DSM-V) [11]. It has been linked with obsessive compulsive disorder (OCD), addictive behaviors, and family stress. These patients often exhibit awareness of obsessive cravings and acts of pica, but are unable to stop the compulsions. Pica occurs in patients of all educational backgrounds, but there is a high prevalence rate in individuals with intellectual disability. As with our patient, symptoms of pica may go undiagnosed for years. A patient may go to extreme measures to hide symptoms from friends and family members and be hesitant to discuss these behaviors with physicians.

In a patient with iron deficiency anemia and recurrent nephrolithiasis, using open-ended, non-judgmental questions such as “Do you have any cravings?” and explaining that “It is common for patients with anemia to experience certain cravings” will increase the likelihood of unmasking previously missed pica [12]. Giving examples of different common pica cravings can be beneficial as a patient may view eating ice chips or salt as benign or normal. If a nutrient deficiency is determined to be the cause of pica, replacing the depleted nutrient tends to reduce the behavior. However, cognitive behavioral therapy and family support may also be needed to completely resolve symptoms [12].

## Conclusions

We described a rare case of sodium chloride pica associated with recurrent nephrolithiasis. It is important that clinicians consider the possible diagnosis of sodium chloride pica in patients with iron deficiency anemia and recurrent nephrolithiasis. Treatment of anemia and resolution of pica may prove effective in preventing future nephrolithiasis. Specific questioning about pica symptoms in patients with iron deficiency anemia and recurrent nephrolithiasis may be helpful diagnostically and therapeutically.
